# The adverse effects of the methoxsalen and ultraviolent A radiation on spermatogenesis in mice

**Published:** 2015-08

**Authors:** Mona Farhadi, Homa Mohseni Kouchesfahani, Abass Shockravi, Mosaeeb Foroozanfar, Kazem Parivar

**Affiliations:** 1 *Department of Mcrobiology, Karaj Branch, Islamic Azad University, Karaj, Iran.*; 2 *Faculty of Chemistry, Kharazmi University, Tehran, Iran.*; 3 *Department of Biology, Kharazmi University, Tehran, Iran.*

**Keywords:** *Methoxsalen*, *UVA*, *Abnormality*, *Spermatogenesis*

## Abstract

**Background:**

Different investigation showed that 5-methoxypsoralen and 8- methoxypsoralen reduce birth rates in the rats.

**Objective:**

In this study we worked out the effect of methoxsalen together with ultraviolent A (UVA) radiation on mature Balb/C mice spermatogenesis.

**Materials and Methods:**

The LD50 standard was determined 160 mg/kg and the UVA dose which causes erythema was calculated 0.046 J/cm2. A sub-lethal dose of 80 mg/kg of methoxsalen solution was injected intrapritoneally to mature mice and after one hour they were exposed to UVA radiation for 20 minutes. Experiments applied included methoxsalen alone, methoxsalen with UVA, UVA alone, sham group (a group received Tween 80), and control group (N=6). In all experimental groups except UVA alone group, injections were carried out, during two consecutive weeks. Serial cross sections (5 µm thickness) were prepared for morphological and histological studies. Tunica albuginea diameter, and number of type A and type B spermatogonia and histological investigation of the testes were measured.

**Results:**

Microscopical and statistical analyses showed significant anomalies among the experimental groups compared to control and sham group. These anomalies included decrease the body weight; increase the relative testis weight; and decrease the number of spermapogonia (type A and B), primary spermatocytes, spermatids and sperms in experimental groups I and II compared to control group. Our results showed the number of spermatozoa in experimental group I was 22.6±2.12, in experimental group II was 33.6±2.05 and in control group was 44.3±2.77 (p<0.05). Moreover in some experimental groups (I and II) shrinkage of seminiferous tubules and release of primary spermatocyte and spermatids were observed to the lumen of them.

**Conclusion:**

It is concluded from the results of this work that treatment with methoxsalen with UVA can damage and disorganize seminiferous tubules and decrease spermatogenic cells.

## Introduction

Psoralens are a class of three rings, heterocyclic and planar compounds that occur naturally as secondary metabolites in various plant species (1, 2, 3, 4) Methoxsalen (8-methoxypsoralen) together with ultraviolet A (UVA) long-wave therapy (PUVA) is known to be a human carcinogen based on sufficient evidence of carcinogenicity from studies in humans. In photo chemotherapy methoxsalen and UVA (320-400 nm) are used for treatment of some epidermal proliferative disorders such as psoriasis and vitiligo (5, 6). Psoralens are known to be toxic to a wide range of organisms (7). In the last three decades researchers were able to show that psoralens inhibit epidermal cell proliferation after UV light irradiation (6). 

It has been shown that methoxsalene has toxic effects on reproductive and developmental processes (8). Studies by Diawara *et al.* (1997) showed that, oral administration of methoxsalen to male and female rats reduces birth rate significantly. Also, 200 to 400 mg/Kg dosage of methoxsalen could reduce animal weight and could change the histology of the liver, testes and adrenal (9). In 2013 it was shown that methoxsalen decreased mean level of estrogen and progesterone significantly and it significantly reduced the number and diameter of corpus luteum and number of growing follicles (10). Ruta graveolens (RG) is one of the species of herbal medicinal families that use for male contraception (11, 12). Long term administration of RG can decrease the weight of genital organs, sperm motility and sexual behavior in rat (13). RG plus 8-methoxy psoralen (sodab) caused weight gain in testicle and epydidim (14). These findings showed that psoralen could affect the male and female reproductive systems. Despite of the long term application of furocoumarines (methoxsalen, xanthotoxin, bergapten, isopimpinellin) in traditional medicine, the teratogenicity of methoxsalen alone or together with UV radiation is not clear yet. This study was applied to investigate the probable teratogenicity of methoxsalen on unmated male mature Balb/C mice.

## Materials and methods

In an experimental study in Kharazmi University we used Methoxsalen with formula of 9-methoxy-7H-furo [3, 2-g] [1]-benzopyran-7-one that was purchased from Sigma Aldrich (Germany) and Tween 80 was used as solvent. The UVA source consisted of a L40/79k (Osram) lamp emitting 7.632×10 (w/cm) at 50 cm distance. The UVA dose which causes erythema is 0.046 J/cm2. Male Balb/C mice were obtain from Pasture Institute (Experimental Animal Keeping Center, Tehran, Iran) and were grown in the animal room at temperature of 22-23^°^C, 50-55% humidity and lighting cycle of 12-h light/12-h dark. The animals were randomly divided in three experimental, sham and control groups which all were unmated mature Balb/C mice (65 days old), 6 in each group. The LD50 standard was determined 160 mg/kg body weight and the UVA dose which causes erythema was applied (0.046 g/cm). A sub-lethal dose of 80 mg/kg methoxsalen solution was injected intrapritoneally to mature mice and after one hour the mice were exposed to UVA radiation for 20 minutes. Experimental group I received methoxsalen only; experimental group II received methoxsalen with UVA and PUVA, while experimental group III were under UVA only. Injections were applied for two consecutive weeks. The animals of experimental groups (I, II, III) were sacrificed 48 hours after the last injection, and their testes were removed and fixed in Bouins’s fixative and embedding with paraffin. Then serial cross sections (5 µm thickness) were prepared and slides were stained with H & E solutions for morphological and histological studies (15). The following observations were recorded: initial and final male body weights, testes weights, Tunica albuginea diameter, and number of spermatogonia and histological investigation of the testes. Morphometric measurements were applied by light microscopy. Tubule diameters of 100 nearly round seminiferous tubules were measured for each testis using an ocular micrometer at 250X. Diameter averaged for each rat for statistical analysis (16). All animal-related protocols were approved by Ethical Committee at Kharazmi University, Tehran, Iran.

**Statistical analysis**

In all experiments, data were analyzed using SPSS (Statistical Package for the Social Sciences, SPSS Inc., Chicago, IL, USA) software, and one way ANOVA statistical method. For each variable, means were calculated at the 5% level using LSD test.

## Results

**Figure 1:**
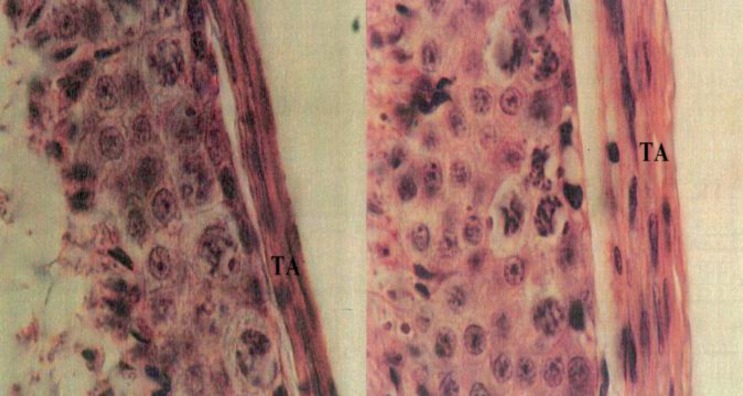
Photomicrograph of tunica albuginea of testis. Control (left), experimental: methoxsalen with UVA (right). The increase of tunica albuginea diameter (2500X)

**Figure 2:**
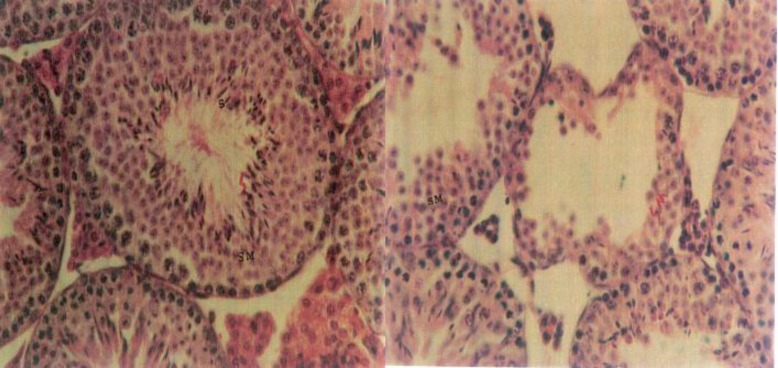
Photomicrograph of seminiferous tubules of testis. Control (left) experimental: methoxsalen with UVA (right). The shrank seminiferous tubule with empty lumen of sperm (1000X

The results showed that body weight reduced significantly in all experimental groups compared to the control group. Relative testes weight and the thickness of tunica albuginea, was significantly increased (Figure 1) in experimental groups I and II, while the testis volume and its length and diameter did not show significant changes (Table I). The number of type A and B spermatogonia, primary spermatocytes, spermatids and sperms were significantly reduced in all experimental groups, compared to control group (Table II). In addition, PUVA experience (experimental group I) and methoxsalen alone expeience (experimental group II) showed shrinkage of seminiferous tubules, release of primary spermatocytes and sperms into the lumen, and disorganized seminiferous tubules (Figure 2).

**Table I:** Results of the effects of methoxsalen on the testes (mean±SE)

**Groups**	**Body weight ** *	**Relative testis weight** **	**Thickness of Tunica albuginea µm**
Controls	2.35±0.57	0.774±0.03	13.7±0.63
Sham	2.17±0.71	0.779±0.03	14.74±0.66
Methoxalen (experimental group I)	-12.51±0.71	0.885±0.03	21.5±0.82
Methoxalen with UVA (experimental group II)	-11.9±1.18	0.883±0.03	31.62±0.84
UVA (experimental group III)	-1.28±0.57	0.771±0.02	19.7±0.77

b: Statistical difference to control group (p<0.05)

UVA: Ultraviolent A

* Body weight gain= (w2-w1)/w1×100: w1=animal weight before experiment, w2=animal weight after experiment and SE=standard error

** Relative testis weight =(testis weight/body weight)×100

**Table II:** Results of the effects of methoxsalen on the number of spermatogenic cells (mean±SE)

**Groups**	**No. of spermatogonia Type A**	**No. of spermatogonia** **Type B**	**No. of primary spermatocyte**	**No. of spermatid**	**No. of ** **spermatozoid**
Controls	2.4±0.12	13.52±0.67	18.2±0.82	66.5±2.81	44.3±2.77
Sham	2.2±0.11	13.22±0.65	17.4±0.79	64.9±2.63	44.6±2.76
Methoxalen (experimental group I)	1.44±0.13	9.6±0.715	15.14±0.73	39.9±2.67	22.6±2.12
Methoxalen with UVA(experimental group II)	1.28±0.11	7.64±0.497	12.04±0.71	46.8±2.84	33.6±2.05
UVA (experimental group III)	1.52±0.12	10.96±0.67	16.7±0.71	49.1±1.54	38.5±2.56

b: Statistical difference to control group (p<0.05).

UVA: Ultraviolent A

## Discussion

8-methoxypesoralen (methoxsalen) with UVA (PUVA) has recovery effect on several autoimmune diseases including psoriatic arthritis, skin disorders and also reproductive system (8, 16). Despite the treatment effects of PUVA, it has photo toxicity, mutagenic and carcinogenic effects on various physiological pathways and cell components (15, 17, 18). Different studies indicated, various byproducts of methoxsalen could have toxicity effects on cell membrane or nucleic acids (19). Our findings showed, methoxsalen can affect male reproductive organs; toxicity of methoxsalen lead to decrease in the number of spermatocytes, spermatids and sperms in all experimental groups compared to control group.

In addition, PUVA induces chromosomal aberrations, sister chromatid exchanges, mutations, damage and cross-links in DNA of human cells in vitro (20). In our study, methoxsalen alone and with PUVA showed significant decrease in the mice body weight. This decrease in the body weight showed that methoxsalen can act as an important repressor for cell division. Thus, the interaction of PUVA with different parts of the cell such as DNA results in decreasing normal development. Investigation of the in vitro interaction between DNA-methoxsalen in darkness resulted in two mechanisms for DNA-methoxsalen binding: in low dosage methoxsalen intercalates with DNA whereas in high dosage methoxsalen remains out of the DNA molecule (21). Our result showed that intraperitoneal administration of 80mg/kg of methoxsalen for two weeks results in significant decrease in body weight and the number of spermatogenic cells specifically spermatids and spermatozoids. This finding could be due to interaction between methoxsalen–DNA which leads to chromosomal aberrations in low dosage and short period of time.

Sufficient evidences for carcinogenicity of methoxsalen and UVA on experimental animals exist. Methoxsalen applied by oral, intraperitoneal administration and skin application in combination with ultraviolet radiation induced epidermal and dermal tumors in the mice (19). Increase in relative testis weight and diameter of tunica albuginea only in experimental groups I and II could be as a results of anti-apoptotic and tumor induction effects of methoxsalen. Whereas, this increase in experimental group III compared to control group was not significant; one reason for this result could be because of accumulative side effects of UVA together with methoxsalen that needs further investigation.

Methoxsalen induces liver enzyme mRNA, cytochrome P4501A1 and UDP-glucuronosyltransferase1A6, suggesting enhancement of metabolism of estrogen. Thus, methoxsalen treatment may explain the reproductive toxicity including reduction of ovarian follicular function and ovulation, effect on pituitary–gonad axis, and ovarian function (9, 10) Mated females treated by methoxsalen require more time to become pregnant and mated males treated by methoxsalen need more breeding attempts (12). This finding showed that the methoxsalen has effects on both males and females. Our results for experimental group I and II showed shrinkage and disorganize in seminiferous tubules and release of primary spermatid cells, while there was not any change in group III. This result indicated the effect of methoxsalen on pituitary axis. Ruta graveolens L. (RG) contains various types of secondary metabolites including coumarins, furanocoumarins (methoxsalen, xanthotoxin, bergapten and isopimpinellin). The studies of RG showed a significant decrease in the number of spermatogonia type A and B, primary spermatatocyte, spermatid, and sperm in the experimental groups as a result of methoxsalen (14). Although these results are compatible with our results, our data were obtained in lower dose and shorter time (80mg/kg and 2 weeks) compared to RG result (300 mg/kg and 100 days). It showed that adverse effects of methoxsalen even start with low dose and in the short time.

Another study showed that treated males Wistar rats had significantly smaller pituitary glands, fewer sperm per ejaculation, and fewer sperm in the vasa defferentia and epididymis than control (9); methoxsalen has specific effect on sperm concentration and reproductive ability by inducing CREM gene expression (13). Our results showed only methoxsalen and PUVA decrease spermatogenic cells and body weight. Thus, effect of methoxsalen on pituitary–gonad axis could result in decrease of spermatogenic cells and seminiferous disorganization. In addition, adverse effects of methoxsalen on nucleic acids, cell division or cell death in all spermatogenic cells could be another reason for decreasing spermatogenic cells. We conclude that both methoxsalen and PUVA have toxic effects on the spermatogenesis and all spermatogenic cells. Significant difference between the numbers of spermatozoa was not observed in groups I and II.

Moreover, our results showed that releases of primary spermatocytes and spermatids into the lumen of seminiferous tubules lead to the adverse effects of methoxsalen on spermatogenesis. Our previous research on effects of methoxsalen on oogenesis showed significant difference in long term treatment compared to short term treatment. For instance, increase in diameter of graafian follicle was observe only in short term treatment (22). Thus, investigating short term and long term effects of methoxsalen on spermatogenesis is another important fact to be considered in future researches. For a better understanding of side effects of methoxsalen on reproductive system, it is important to investigate whether the toxic effects of methoxsalen on spermatogenesis is temporary or permanent. This is essential to understand if methoxsalen side effects will reverse when the treatment has stopped. More studies about exact mechanism (s) of this compound on Sertoli cells are needed.

## Conclusion

We conclude, that administration of methoxsalen together with UVA radiation can damage the spermatogenic germ cells in mice, disorganize seminiferous tubules, decrease spermatogenic cells, decrease body weight and increase diameter of tunica albuginea.
